# Left ventricular hypertrophy and incident cognitive decline in older adults with hypertension

**DOI:** 10.1038/s41371-022-00681-1

**Published:** 2022-04-01

**Authors:** Ying Xu, George Bouliotis, Nigel S. Beckett, Riitta L. Antikainen, Craig S. Anderson, Christopher J. Bulpitt, Ruth Peters

**Affiliations:** 1 Neuroscience Research Australia, Margarete Ainsworth Building, Barker Street, Randwick, NSW 2031 Australia; 2 University of New South Wales, Sydney, NSW 2052 Australia; 3 Warwick Clinical Trials Unit, Warwick Medical School, University of Warwick, Coventry, UK; 4 Guys and St Thomas’ NHS Trust, London, UK; 5 Centre for Life-Course Health Research/Geriatrics, University of Oulu, Oulu, Finland; 6 Medical Research Centre Oulu, Oulu University Hospital, Oulu, Finland; 7 The George Institute for Global Health, Level 5, 1 King St, Newtown, NSW 2042 Australia; 8 The George Institute for Global Health at Peking University Health Science Centre, Level 18, Tower B, Horizon Tower, No. 6 Zhichun Rd, Haidian District, Beijing, 100088 P.R. China; 9 Neurology Department, Sydney Local Area Health District, Royal Prince Alfred Hospital, Camperdown, NSW 2050 Australia; 10 Imperial College London, London, W2 1PG UK

**Keywords:** Hypertension, Risk factors

## Abstract

The association between raised blood pressure and increased risk of subsequent cognitive decline is well known. Left ventricular hypertrophy (LVH), as a marker of hypertensive target organ damage, may help identify those at risk of cognitive decline. We assessed whether LVH was associated with subsequent cognitive decline or dementia in hypertensive participants aged ≥80 years in the randomized, placebo-controlled Hypertension in the Very Elderly Trial. LVH was assessed using 12-lead electrocardiography (ECG) based on the Cornell Product (CP-LVH), Sokolow-Lyon (SL-LVH), and Cornell Voltage (CV-LVH) criteria. The Mini-Mental State Examination (MMSE) was used to assess cognitive function at baseline and annually. A fall in MMSE to <24 or an annual fall of >3 points were defined as cognitive decline and triggered dementia screening (Diagnostic Statistical Manual IV). Death was defined as a competing event. Fine-Gray regression models were used to examine the relationship between baseline LVH and cognitive outcomes. There were 2645 in the analytical sample, including 201 (7.6%) with CP-LVH, 225 (8.5%) SL-LVH and 251 (9.5%) CV-LVH. CP-LVH was associated with increased risk of cognitive decline, subdistribution hazard ratio (sHR)1.3 (95% confidence interval (CI) 1.01–1.67) in multivariate analyses. SL-LVH and CV-LVH were not associated with cognitive decline (sHR1.06 (95% CI 0.82–1.37) and sHR1.13 (95% CI 0.89–1.43), respectively). LVH was not associated with dementia. LVH may be related to subsequent cognitive decline, but evidence was inconsistent depending on ECG criterion and there were no associations with incident dementia. Additional work is needed to understand the relationships between blood pressure, LVH assessment and cognition.

## Introduction

Although it is established that the degree and duration of exposure to raised blood pressure (BP) are inversely associated with cognitive performance [1, 2], the impact of raised BP on risk of cognitive decline and dementia appears strongest when experienced in midlife with the evidence in later life more mixed [3, 4]. Left ventricular hypertrophy (LVH), as a primary target for hypertensive end-organ damage, reflects the magnitude and chronicity of BP elevation and thus may be able to identify a particular older adult population at risk, i.e. those with a longer history of raised BP [5]. LVH has also been associated with poorer cognitive performance in meta-analyses of five longitudinal and four cross-sectional studies (*n* = 28,648) where those with electrocardiogram (ECG) or echocardiography assessed LVH were 1.4 times (odds ratio (OR), 95% confidence interval (CI) 1.18–1.66) more likely to have cognitive impairment [6]. Meta-analyses of the three cross-sectional studies that included only hypertensive participants (*n* = 1262) found that LVH was associated with double the risk of cognitive impairment (OR 2.14, 95% CI 1.39–3.30) [6]. However, only four out of these 12 studies used ECG [7–10], which is listed in guidelines as required for routine assessment for LVH in all hypertensive patients [11, 12].

Ageing is independently involved in left ventricular remodelling [13], and globally the prevalence of dementia increases with ageing [14]. However, to our knowledge, there are only three studies examining ECG defined LVH and its relationship with subsequent cognitive decline in those aged 70 years old or over [8, 9, 15]. Furthermore, these three studies report conflicting results [8, 9, 15], and no study has yet examined older adults with hypertension. In this study, we assessed whether ECG criteria defined LVH were associated with the risk of developing cognitive decline and dementia in participants in the Hypertension in the Very Elderly Trial (HYVET) [16, 17].

## Subjects and methods

HYVET was a double-blind, randomized, placebo controlled trial of indapamide 1.5 mg sustained release or matching placebo with the optional addition of 2 or 4 mg perindopril, or matching placebo to reach target BP of <150/80 mmHg (ClinicalTrials.gov number: NCT00122811) [16, 17]. Participants were older adults (aged ≥ 80 years old) with a mean seated systolic BP (SBP) between 160 and 199 mmHg, a mean standing SBP ≥ 140 mmHg, and a mean seated diastolic BP (DBP) < 110 mmHg [16, 17]. Exclusion criteria included contraindication to trial medication, accelerated hypertension, secondary hypertension, hemorrhagic stroke in the previous 6 months, heart failure requiring treatment with antihypertensive medication, serum creatinine level > 150 mmol/l, serum potassium level < 3.5 mmol/l or >5.5 mmol/l, gout, a clinical diagnosis of dementia and requirement for nursing home care, etc [16, 17]. HYVET was conducted in 195 centers in 13 countries. Ethical approvals were obtained, and all participants gave written informed consent. As the trial reported no statistically significant relationship between trial treatment and cognitive decline or dementia [18], the HYVET data were essentially treated as a cohort, albeit with adjustment for randomised group allocation for the purposes of these analyses.

### Left ventricular hypertrophy (LVH) and ECG measurements

Two clinical doctors (NSB and RLA) evaluated the baseline resting 12-lead ECG [17], and made an assesment of LVH based on three criteria. Details of the ECG assessment have been published previously [17]. In brief, LVH was considered as present by Cornell product criterion (CP-LVH) when the QRSd multiplied by (RaVL + SV3) was above 244 mVms for males and the QRSd multiplied by (RaVL + SV3 + 0.6) was above 244 mVms for females. LVH was considerred as present by Sokolow-Lyon voltage (SL-LVH) criterion when the amplitude of SV1 + (RV5 or RV6, whichever is larger) was above 3.8 mV, and by Cornell voltage criterion (CV-LVH) when the amplitude of RaVL + SV3 was above 2.8 mV for males and above 2.0 mV for females. These LVH measures were additionally explored as continuous variables. The presence of bundle branch block was defined as a QRSd ≥ 120 ms.

### Cognitive decline, dementia and competing outcomes

Cognitive function was assessed at baseline and annually thereafter using the Mini-Mental State Exam (MMSE). A reduction in MMSE score to below 24 or by more than three points in one year was classified as cognitive decline and triggered a dementia assessment [19]. Dementia was diagnosed based on the Diagnostic Statistical Manual IV [19]. An independent expert dementia committee blind to trial treatment allocation reviewed and validated all dementia endpoints. Death from any cause, one of the trial endpoints, was defined as a competing event in the current study. A trial endpoint committee comprised of international experts blind to trial treatment allocation validated trial endpoints, based on supporting documentation, e.g. death certificates and hospitalization reports.

### Statistical analyses

The difference in baseline characteristics between those who were included in the analytical sample and those who were excluded, and between those with and without LVH, were assessed using Chi-squared, *t* tests and Wilcoxon tests, as appropriate. We used Fine-Gray regression models, examining the relationship between LVH status and subsequent cognitive decline or dementia taking competing events (death) into account, where participants not experiencing cognitve decline or dementia but experiencing the competing event (death) were treated as being censored at infinity to indicate that they would never experience the event of interest [20]. The date of the study visit where cognitive decline was identified was taken as the date of cognitive decline and as the date of dementia if so diagnised.

Propotional hazard assumption was checked by fitting a time-dependent covariate and checking Schoenfeld residuals. Potential confounders were adjusted for or stratified, as required to meet propotional hazard assumptions. These included the trial treatment (placebo versus antihypertensive treatment) and baseline characteristics: age, sex, education (any versus no formal education), SBP, DBP, serum cholesterol level, body mass index (BMI, calculated using measured weight and height, kg/m^2^), and presence or absence of the following variables: previous treatment for hypertension, atrial fibrillation defined as self-reported and/or via ECG evidence, cardiovascular disease, diabetes mellitus, current smoking and alcohol consumption. Diabetes was defined as reported diabetes, or in receipt of antidiabetic treatment, or a random blood glucose measurement of >11.1 mmol/l. These variables were selected as those that have been shown to be related to the risk of dementia [21]. Effect modification due to sex and trial medication were examined. In sensitivity analyses, models were repeated in those with baseline MMSE ≥ 24, whom are considered as unlikely to have pre-existing cognitive decline and/or undiagnosed dementia. Inverse probability weighting was used to evaluate the potential impact of attrition. Due to the insidious nature of cognitive decline onset and individuals‘ differences in the number of MMSE tests and spacing between tests, dates of events used in the analyses can be inaccurate. Therefore, multinomial logistic regression taking account of death as a competing risk was used as a more conservative option in the sensitivity analyses. All statistical analyses were carried out using SAS version 9.4. Significance was set at *p* < 0.05, and all tests were two-tailed.

### Study sample

There were 3845 participants in the HYVET trial, of whom 1200 were excluded from the current analyses due to missing, uncodable, incomplete ECG or ECG with bundle branch block, no cognitive assessment, or missing information on covariates (Fig. e-1). In sensitivity analyses, the subset of those who had baseline MMSE ≥ 24 included 1836 participants.

## Results

The analytical sample included 2645 participants with a mean follow-up of 2 years (standard deviation (SD) 1.3 years) for cognitive decline, and of 2.2 years (SD 1.4 years) for dementia. The analytical sample was younger, more likely to be female, and less likely to have atrial fibrillation or history of cardiovascular disease, compared to those who were excluded (Table e-1, all *p* < 0.05). They also had higher DBP (all *p* < 0.05), but otherwise did not differ from those who were excluded.

Among the 2645 participants in the analytical sample, 201 (7.6%) had CP-LVH, 225 (8.5%) had SL-LVH, and 251 (9.5%) had CV-LVH. When examining the characteristics of those classified as having LVH compared to those without, in general, those with LVH had higher SBP but other characteristics varied depending on the classification used. Specifically, those who had CP-LVH were older, more likely to be female, to have formal education, higher SBP and DBP, and greater BMI, and to have atrial fibrillation (all *p* < 0.05) than those who did not have CP-LVH (Table 1). Those classified as having SL-LVH by contrast had higher SBP, but had lower values of BMI and cholesterol (all *p* < 0.05) than those who did not have SL-LVH. Those who had CV-LVH were more likely to be female, to have formal education, to have atrial fibrillation, and less likely to use alcohol and to be current smoker (all *p* < 0.05), than those who did not have CV-LVH. They also had higher SBP and DBP and greater BMI (all *p* < 0.05).

**Table 1:** Characteristics by left ventricular hypertrophy status (*n* = 2645).

Baseline characteristics, unless specified	Left ventricular hypertrophy status accessed based on								
	Cornell product criterion			Sokolow-Lyon criterion			Cornell voltage criterion		
	Present (*n* = 201, 7.6%)	Absent (*n* = 2444)	*P* value	Present (*n* = 225, 8.5%)	Absent (*n* = 2420)	*P* value	Present (*n* = 251, 9.5%)	Absent (*n* = 2394)	*P* value
Age in years	83 (81.4, 86.2)	82.3 (81.1, 85)	**0.005**	82.6 (81.3, 85.4)	82.4 (81.1, 85.1)	0.18	82.6 (81.1, 85.5)	82.4 (81.1, 85.1)	0.23
Aged ≥85 years old	65 (32.3)	619 (25.3)	**0.03**	66 (29.3)	618 (25.5)	0.21	71 (28.3)	613 (25.6)	0.36
Female	159 (79.1)	1484 (60.7)	**<0.0001**	129 (57.3)	1514 (62.6)	0.12	223 (88.8)	1420 (59.3)	**<0.0001**
Formal education	165 (82.1)	1760 (72)	**0.002**	162 (72)	1763 (72.9)	0.78	204 (81.3)	1721 (71.9)	**0.002**
SBP mmHg	173 (168.5, 180)	171.3 (166.5, 177.8)	**0.001**	172.5 (167.5, 181)	171.5 (166.5, 177.8)	**0.007**	173 (168.5, 181)	171.3 (166.5, 177.5)	**<0.0001**
DBP mmHg	94 (90.5, 97.5)	93 (85.5, 96.6)	**0.004**	93 (85.3, 97.5)	93 (86, 96.8)	0.84	94 (90, 97.8)	93 (85.5, 96.5)	**0.002**
DBP < 90 mmHg	43 (21.4)	838 (34.3)	**0.0002**	75 (33.3)	806 (33.3)	0.99	62 (24.7)	819 (34.2)	**0.002**
Cholesterol mmol/L	5.4 ± 1.2	5.3 ± 1.1	0.45	5.1 ± 1.1	5.3 ± 1.1	**0.0005**	5.4 ± 1.2	5.3 ± 1.1	0.58
BMI kg/m^2^	25.3 ± 4.1	24.6 ± 3.6	**0.04**	23.4 ± 3.4	24.8 ± 3.7	**<0.0001**	25.3 ± 4	24.6 ± 3.6	**0.02**
Antihypertensive treatment	132 (65.7)	1560 (63.8)	0.60	152 (67.6)	1540 (63.6)	0.24	169 (67.3)	1523 (63.6)	0.24
Atrial fibrillation	22 (11)	149 (6.1)	**0.007**	13 (5.8)	158 (6.5)	0.66	28 (11.2)	143 (6)	**0.002**
History of CVD	23 (11.4)	262 (10.7)	0.75	25 (11.1)	260 (10.7)	0.87	26 (10.4)	259 (10.8)	0.82
Diabetes	22 (11)	239 (9.8)	0.59	21 (9.3)	240 (9.9)	0.78	32 (12.8)	229 (9.6)	0.11
Current smoker	9 (4.5)	162 (6.6)	0.23	17 (7.6)	154 (6.4)	0.49	8 (3.2)	163 (6.8)	**0.03**
Use of alcohol	31 (15.4)	441 (18)	0.35	37 (16.4)	435 (18)	0.57	32 (12.8)	440 (18.4)	**0.03**
Treatment vs placebo	108 (53.7)	1237 (50.6)	0.40	113 (50.2)	1232 (50.9)	0.84	136 (54.2)	1209 (50.5)	0.27
MMSE	26 (23, 28)	26 (23, 28)	0.55	26 (22, 28)	26 (23, 28)	0.65	26 (23, 28)	26 (23, 28)	0.87
Number of MMSE measures	3.6 ± 1.6	3.4 ± 1.5	0.11	3.3 ± 1.6	3.4 ± 1.5	0.64	3.6 ± 1.6	3.4 ± 1.5	**0.02**

Numbers are mean ± standard deviation for continuous variables with normal distribution, median (Q1 lower quartile, Q3 upper quartile) for continuous variables with skewed distribution, or *n* (%) for categorical variables.

Statistically significant *P* values are in bold.

*BMI* denotes body mass index, *CVD* cardiovascular disease, *DBP* diastolic blood pressure, *MMSE* Mini-Mental State Examination, *MI* myocardial infarction, *SBP* systolic blood pressure.

### CP-LVH

After adjusting for age, sex, SBP, cholesterol level, BMI, previous treatment for hypertension, presence or absence of atrial fibrillation, cardiovascular disease, diabetes mellitus, current smoking status, alcohol consumption, and trial treatment, and stratified by education and diastolic hypertension status, CP-LVH was associated with increased risk of developing cognitive decline, subdistribution Hazard Ratio (sHR) 1.3 (95% confidence interval (CI) 1.01–1.67, *p* = 0.04, Table 2, Fig. e-2). Statification was used for DBP and education as these variables failed the proportional hazards assumptions, and DBP was categorised as having diastolic hypertension (DBP ≥ 90 mmHg) versus not having diastolic hypertension (DBP < 90 mmHg). In sensitivity analyses, when including only those who had baseline MMSE ≥ 24, *n* = 1836), the associations became stronger (Table 2). There was no association between CP-LVH and the risk of developing dementia in any analyses, however point estimates were consistently greater than unity in adjusted models. When CP-LVH measure was used as a continuous variable, it showed the same pattern of associations for both cognitive decline and dementia (Table e-2).

**Table 2:** Associations between left ventricular hypertrophy and incident cognitive decline or dementia in the Fine-Gray regression models.

		Cognitive decline Reduction in MMSE score to <24 or by >3 points in a year				Dementia			
		Analytical sample (*n* = 2645)		Among those who had baseline MMSE of ≥24 (*n* = 1836)		Analytical sample (*n* = 2645)		Among those who had baseline MMSE of ≥24 (*n* = 1836)	
		Cognitive decline (*n* = 785) Competing events (*n* = 111)		Cognitive decline (*n* = 569) Competing events (*n* = 74)		Dementia (*n* = 225) Competing events (*n* = 164)		Dementia (*n* = 179) Competing events (*n* = 109)	
		sHR (95% CI)	*P* value	sHR (95% CI)	*P* value	sHR (95% CI)	*P* value	sHR (95% CI)	*P* value
CP	Uni-	1.25 (0.98, 1.59)	0.07	**1.35 (1.03, 1.78)**	**0.03**	0.88 (0.54, 1.44)	0.60	0.94 (0.54, 1.61)	0.81
	Multi-	**1.3 (1.01, 1.67)**	**0.04**	**1.45 (1.08, 1.93)**	**0.01**	1.03 (0.63, 1.7)	0.90	1.15 (0.66, 2.01)	0.63
SL	Uni-	1.06 (0.83, 1.36)	0.62	1.05 (0.79, 1.39)	0.76	1.08 (0.68, 1.71)	0.75	0.95 (0.56, 1.62)	0.86
	Multi-	1.06 (0.82, 1.37)	0.65	1.07 (0.8, 1.44)	0.65	1.17 (0.72, 1.88)	0.53	1.09 (0.63, 1.88)	0.76
CV	Uni-	1.11 (0.88, 1.39)	0.39	1.14 (0.88, 1.48)	0.32	1.12 (0.75, 1.69)	0.58	1.21 (0.78, 1.89)	0.39
	Multi-	1.13 (0.89, 1.43)	0.32	1.17 (0.89, 1.53)	0.26	1.32 (0.87, 2.01)	0.19	1.45 (0.91, 2.29)	0.12

*CI* confidence interval, *CP* Cornell product criterion, *CV* Cornell voltage criterion, *MMSE* Mini-Mental State Examination, *Multi-* multivariate analyses, *sHR* subdistribution hazard ratio, *SL* Sokolow-Lyon voltage criterion, *Uni-* univariate analyses.

Statistically significant sHRs and *P* values are in bold.

For the outcome of cognitive decline, multivariate models were adjusted for age, sex, systolic blood pressure, cholesterol level, body mass index, previous treatment for hypertension, atrial fibrillation, cardiovascular disease, diabetic mellitus, current smoking status, alcohol consumption, and trial treatment (placebo vs antihypertensive treatment), and stratified by education (formal vs non-formal) and diastolic blood pressure (<90 mmHg vs ≥90 mmHg).

For the outcome of dementia, multivariate models were adjusted for sex, education (formal vs non-formal), systolic blood pressure, cholesterol level, body mass index, atrial fibrillation, cardiovascular disease, diabetic mellitus, current smoking status, alcohol consumption, and trial treatment (placebo vs antihypertensive treatment), and stratified by age (<85 vs ≥85 years old, diastolic blood pressure (<90 mmHg vs ≥90 mmHg) and previous treatment for hypertension.

### SL-LVH and CV-LVH

There were no associations between SL-LVH and the risk of incident cognitive decline or dementia (Table 2) although point estimates were consistent with the CP-LVH results. Similarly for CV-LVH, with the exception of CV-LVH examined as a continuous variable, where it showed an association with increased risk of cognitive decline in sensitivity analyses among those with baseline MMSE ≥ 24 (*p* = 0.04, Table e-2).

### Effect modification, attrition, and multinomial logistic regression

We found no effect modification by sex and trial treatment in any of the models. Using inverse probability weighting to examine the potential impact of attrition (Table e-3), and multinomial logistic regression to account for the likely inaccuracy in event dates (Table e-4) showed similar results.

## Discussion

LVH was identified in 7.6%, 8.5% and 9.5% of the study sample using CP-LVH, SL-LVH and CV-LVH criteria, respectively. Overall the pattern of results was consistent although statistically significant only for the outcome of cognitive decline and CP-LVH and to a lesser extent CV-LVH.

Other longitudinal studies that have reported on ECG defined LVH and cognitive outcomes in older adults, have also reported mixed findings. In an American population assessed in 1980s, older adults (average age 79 years) showed no association between LVH (criterion unclear) and incident dementia (Diagnostic Statistical Manual III) [9]. In the Helsinki Aging Study which included three birth cohorts aged 75, 80 and 85 years old, the presence of LVH was not associated with decrease of over 3 points in MMSE or increase in Clinical Dementia Rating during follow-up [8]. Finally, in the PROspective Study of Pravastatin in the Elderly at Risk (PROSPER) trial, a higher baseline Sokolow-Lyon product index was associated with steeper decline in selective attention, processing speed, and immediate and delayed memory over a mean follow-up of 3.2 years among 5804 participants aged 70 to 82 years, after adjustment for cardiovascular risk factors, co-morbidities and medications [15]. However, the Sokolow-Lyon product index was used as a continuous variable in this study, which limits the clinical meaningfulness [15].

In contrast, longitudinal studies in younger populations have consistently reported associations between ECG criteria defined LVH and cognitive outcomes. Firstly, in the Whitehall II study, in a study sample at a mean age of 55.6 (SD 6) years old with LVH assessed using the Minnesota code [22], they found that LVH was associated with greater decline in a subsequent 10-year change in a composite score of reasoning, memory, phonemic and semantic fluency and vocabulary (*p* = 0.05) [22]. Similarly, the Reasons for Geographic and Racial Differences in Stroke (REGARDS) study (mean age 64.3 (SD 9.2)), found that CV-LVH was related to the development of cognitive impairment (decline from a Six-Item Screener score of ≥5 to a follow-up score of ≤4), OR 1.29 (95% CI 1.06–1.58) during a mean follow-up of 4.1 years [10]. Finally, the Atherosclerosis Risk in Communities (ARIC) study (mean age 56.9 (SD 5.7) years) [23] found CV criterion defined LVH was associated with a higher risk of dementia HR 1.90 (95% CI 1.47–2.44) during a mean follow-up of 18 years [23].

The general lack of a strong association between LVH and cognitive decline or dementia in HYVET may be due to a shorter follow-up (an average of 2 to 2.2 years in the current study compared to longer follow-up in the studies of younger adults) and the use of a screening test to define cognitive decline rather than neuropsychological tests. We note the wide confidence intervals, with point estimates being greater than unity in general. In addition, our participants were older adults. On the one hand this may have meant that they were more likely to have confounding health conditions and to be a population among whom the association between LVH and cognitive outcomes may therefore be weakened. On the other hand, as is frequently the case for clinical trials, the HYVET participants were likely to have been healthier than their counterparts in general population, as evidenced by the low prevalence of previous cardiovascular disease at baseline [16]. Older adults, who survived longer after developing LVH at younger age or who developed LVH in older age, may also be less vulnerable to adverse prognoses. Furthermore, despite the potential for LVH to identify those with a longer term exposure to raised BP and therefore potentially to select those who had experienced raised BP from mid into later life, our results are congruent with the literature on BP and risk of cognitive decline and dementia in older adults where the relationship seems to be attenuated compared to midlife [8, 9, 23]. Lastly, the reported association between CP-LVH and cognitive decline was after adjustment for systolic and diastolic blood pressure BP. However, the fact that in over 30% of hypertensive patients, brain damage (e.g. stroke, cerebral small vessel disease, memory loss) can be the only hypertensive target organ damage [24], may have contributed to the lack of strong associations.

It should also be noted that we found only CP-LVH to be consistently associated with cognitive decline and the accuracy of ECG defined LVH criteria may need to be taken into account. Although a 1990’s study using autopsy as a gold-standard for diagnosis of LVH reported CP having a specificity of 95% and identifying LVH with greater sensitivity than CV (51% versus 36%) [25] a more recent study has reported similar sensitivity and specificity for CP and CV in detecting magnetic resonance imaging defined LVH, whereas Sokolow-Lyon criterion had a higher sensitivity but a lower specificity [26]. When assessed by echocardiography, combined LVH prevalence was 36% among adults with hypertension aged 44–67 years in a systematic review [27]. The prevalence of LVH in our study was less than a-third of that number. Notably, comparing the point estimates for odds ratios, greater increased risk of cognitive impairment among individuals with LVH than without LVH was found when assessing LVH by echocardiography than ECG [6]. Therefore, although echocardiography is less affordable as a routine basic screening test, it may be a better assessment tool for LVH to predict cognitive function.

A strength of our analyses is the use of a well characterized older adult population with hypertension, aged ≥80 years, with a SBP of ≥160 mmHg, and prospective data collection on cognitive decline and dementia. The use of such a clinical trial population also brings the potential for limitations due to selection bias, e.g. those who had a contraindication to trial medications, those with accelerated or secondary hypertension or gout, and those who required nursing care were excluded from the recruitment. Secondly, our definition of cognitive decline was based on a screening test and thus is not the same as a clinical diagnosis of mild cognitive impairment, furthermore we cannot evaluate any potential associations between LVH and specific cognitive domains that may be disproportionately influenced by vascular risk, e.g. executive function. Additionally, baseline MMSE scores were similar between those with and without LVH. The standard study pre-specified definition of cognitive decline, which triggered a dementia assessment is unlikely to provide a precise incidence of cognitive problems, as the requirement of a drop to <24 may have missed out dementia cases (for example, other research has shown a maximum score of 29 reported among nursing home residents with dementia [28]) although the annual decline of over 3 ameliorates this to some extent. Thirdly, we do not have information on all potential confounding factors, e.g. anticoagulation, hypertensive mediated brain damage. Finally, since we do not have longitudinal ECG data, we cannot comment on whether LVH was regressed or recovered after antihypertensive medication use which might lead to reduction of cognitive dysfunction, although no effect modification of treatment arm was shown.

## Conclusions and implications

LVH, a long-term consequence of elevated BP, assessed using ECG, may not be useful as a simple and efficient screening tool to identify older adults with hypertension at short term risk of developing cognitive decline or dementia. However, it may add to the broader clinical picture when assessing older adults at risk of cognitive decline and further work is clearly needed to evaluate the prognostic implications of LVH for cognition, over time, in this age group and at younger ages and, alongside this, to assess the implications of using the differing definitions of LVH.

## Summary

### What is known about topic

- Raised blood pressure and cumulative higher pressures are associated with an increased risk of cognitive decline and dementia.
- Left ventricular hypertrophy (LVH), reflecting the magnitude and duration of blood pressure elevation, may be a useful biomarker for a population at particular risk of subsequent cognitive decline and dementia.
- There is little data available in the older adult population, i.e. those who are arguably at greatest risk.

### What this study adds

- In a cohort of older adults with hypertension, LVH at baseline was associated with an increased risk of incident cognitive decline but not incident dementia over a mean follow up of two years.
- LVH as an indicator of cumulative raised blood pressure may be a useful biomarker for subsequent cognitive decline, however, further work is needed to validate and confirm this relationship.

## Supplementary information

Supplementary

## Data Availability

Additional data are available from the corresponding author on reasonable request.
