# Supplementary material for: Left ventricular hypertrophy and incident cognitive decline in older adults with hypertension
Source: J Hum Hypertens. 2022 Apr 1;37(4):307–12. doi: 10.1038/s41371-022-00681-1 (PMC10063439; doi:10.1038/s41371-022-00681-1)
Supplement: Supplementary file 1 — Supplementary [file 41371_2022_681_MOESM1_ESM.docx]

# Supplementary

This supplementary has been provided by the authors to give readers additional information about their work.

Supplement to: Xu Y, Bouliotis G, Beckett NS, et al. Left ventricular hypertrophy and incident cognitive decline or dementia in elderly hypertensive patients

This supplementary contains the following Tables and Figures:

Table e-1 Baseline characteristics of the analytical sample and those who were excluded

Table e-2 Associations between left ventricular hypertrophy examined as continuous variables and incident cognitive decline or dementia

Table e-3 Associations between left ventricular hypertrophy and incident cognitive decline or dementia after inverse probability weighting account for attrition

Table e-4 Associations between left ventricular hypertrophy and incident cognitive decline or dementia multinomial logistic regression

Figure e-1 Flow chart of the analytical sample and those included in the sensitivity analyses

Figure e-2 Cumulative incidence of cognitive decline by electrocardiography Cornell product criterion defined left ventricular hypertrophy status

# Table e-1 Characteristics of the analytical sample and those who were excluded

| Baseline, unless specified | Analysed (n = 2645) | | Excluded (n = 1200) | P value |
| --- | --- | --- | --- | --- |
| Age in years, median (Q1, Q3) | 82.4 (81.1, 85.1) | 82.7 (81.3, 85.5) | | **0.006** |
| Aged ≥ 85 years old, n (%) | 684 (25.9) | 354 (29.5) | | **0.02** |
| Female vs male, n (%) | 1643 (62.1) | 683 (56.9) | | **0.002** |
| Formal vs no formal education, n(%) | 1925 (72.8) | 890 (74.2) | | 0.37 |
| SBP mmHg, median (Q1, Q3) | 171.5 (166.5, 178) | 171.5 (165.6, 179) | | 0.48 |
| DBP mmHg, median (Q1, Q3) | 93 (86, 97) | 92.5 (85, 96.3) | | **0.02** |
| DBP < 90 mmHg, n (%) | 881 (33.3) | 401 (33.4) | | 0.95 |
| Cholesterol mmol/L, mean (SD) | 5.3 ± 1.1 | 5.3 ± 1.1 | | 0.27 |
| Body mass index kg/m^2^, mean (SD) | 24.7 ± 3.7 | 24.7 ± 3.8 | | 0.81 |
| Antihypertensive treatment, n (%) | 1692 (64) | 794 (66.2) | | 0.19 |
| Atrial fibrillation | 171 (6.5) | 193 (16.1) | | **<0.0001** |
| History of CVD, n (%) | 285 (10.8) | 167 (13.9) | | **0.005** |
| Diabetic mellitus, n (%) | 261 (9.9) | 127 (10.6) | | 0.49 |
| Current smoker, n (%) | 171 (6.5) | 82 (6.8) | | 0.67 |
| Use of alcohol, n (%) | 472 (17.8) | 209 (17.4) | | 0.75 |
| Treatment vs placebo arm, n (%) | 1345 (50.9) | 588 (49) | | 0.29 |
| MMSE, median (Q1, Q3) | 26 (23, 28) | 26 (23, 29) | | 0.95 |
| Number of MMSE measures | 3.4 ± 1.5 | 3.4 ± 1.4 | | 0.58 |

T-tests and Kruskal-Wallis tests were used for continuous variables with normal and skewed distribution, respectively. Chi-squared tests were used for categorical variables.

CVD denotes cardiovascular disease, DBP diastolic blood pressure, MMSE Mini-mental state examination, Q1 lower quartile, Q3 upper quartile, SBP systolic blood pressure, SD standard deviation.

# Table e-2 Associations between left ventricular hypertrophy examined as continuous variables and incident cognitive decline or dementia

|  | | Cognitive decline  Reduction in MMSE score to < 24 or by > 3 points in a year | | | | | | Dementia | | | | | |
| --- | --- | --- | --- | --- | --- | --- | --- | --- | --- | --- | --- | --- | --- |
|  | |  | Analytical sample (n=2645) | |  | Among those who had baseline MMSE of ≥ 24 (1836) | |  | Analytical sample (n=2645) | |  | Among those who had baseline MMSE of ≥ 24 (n = 1836) | |
|  |  |  | Cognitive decline (n=785)  Competing events (n=111) | |  | Cognitive decline (n=569)  Competing events (n=74) | |  | Dementia (n=225)  Competing events (n=164) | |  | Dementia (n=179)  Competing events (n=109) | |
|  |  |  | HR (95% CI) | P value |  | HR (95% CI) | P value |  | HR (95% CI) | P value |  | HR (95% CI) | P value |
| CP | Uni- |  | 1.01 (1, 1.02) | 0.08 |  | **1.01 (1, 1.02)** | **0.04** |  | 1 (0.98, 1.02) | 0.77 |  | 1.01 (0.98, 1.03) | 0.62 |
|  | Multi- |  | **1.01 (1, 1.02)** | **0.05** |  | **1.02 (1, 1.03)** | **0.02** |  | 1.01 (0.99, 1.03) | 0.21 |  | 1.01 (0.99, 1.04) | 0.24 |
| SL | Uni- |  | 1.01 (1, 1.01) | 0.06 |  | 1.01 (1, 1.01) | 0.23 |  | 1.01 (0.99, 1.02) | 0.38 |  | 1 (0.98, 1.02) | 0.90 |
|  | Multi- |  | 1.01 (1, 1.02) | 0.06 |  | 1.01 (1, 1.02) | 0.17 |  | 1.01 (0.99, 1.02) | 0.29 |  | 1 (0.98, 1.02) | 0.99 |
| CV | Uni- |  | 1.01 (1, 1.02) | 0.22 |  | 1.01 (1, 1.02) | 0.24 |  | 1.01 (0.99, 1.03) | 0.62 |  | 1 (0.98, 1.03) | 0.70 |
|  | Multi- |  | 1.01 (1, 1.02) | 0.06 |  | **1.01 (1, 1.03)** | **0.04** |  | 1.01 (0.99, 1.03) | 0.27 |  | 1.01 (0.99, 1.04) | 0.30 |

CI denotes confidence interval, CP Cornell product criterion, CV Cornell voltage criterion, MMSE Mini-Mental State Examination, Multi- multivariate analyses, sHR subdistribution hazard ratio, SL Sokolow-Lyon voltage criterion, Uni- univariate analyses.

For the outcome of cognitive decline, multivariate models were adjusted for age, sex, systolic blood pressure, cholesterol level, body mass index, previous treatment for hypertension, atrial fibrillation, cardiovascular disease, diabetic mellitus, current smoking status, alcohol consumption, and trial treatment (placebo versus antihypertensive treatment), and stratified by education (formal versus non-formal) and diastolic blood pressure (<90mmHg vs ≥90mmHg).

For the outcome of dementia, multivariate models were adjusted for sex, education (formal versus non-formal), systolic blood pressure, cholesterol level, body mass index, atrial fibrillation, cardiovascular disease, diabetic mellitus, current smoking status, alcohol consumption, and trial treatment (placebo versus antihypertensive treatment), and stratified by age (<85 vs ≥85 years old, diastolic blood pressure (<90mmHg vs ≥90mmHg) and previous treatment for hypertension.

# Table e-3 Associations between left ventricular hypertrophy and incident cognitive decline or dementia after inverse probability weighting account for attrition

|  | | Cognitive decline  Reduction in MMSE score to < 24 or by > 3 points in a year | | | | | | Dementia | | | | | |
| --- | --- | --- | --- | --- | --- | --- | --- | --- | --- | --- | --- | --- | --- |
|  | |  | Analytical sample (n=2645) | |  | Among those who had baseline MMSE of ≥ 24 (1836) | |  | Analytical sample (n=2645) | |  | Among those who had baseline MMSE of ≥ 24 (n = 1836) | |
|  |  |  | Cognitive decline (n=785)  Competing events (n=111) | |  | Cognitive decline (n=569)  Competing events (n=74) | |  | Dementia (n=225)  Competing events (n=164) | |  | Dementia (n=179)  Competing events (n=109) | |
|  |  |  | HR (95% CI) | P value |  | HR (95% CI) | P value |  | HR (95% CI) | P value |  | HR (95% CI) | P value |
| CP | Uni- |  | **1.28 (1.06, 1.56)** | **0.01** |  | **1.4 (1.12, 1.75)** | **0.003** |  | 0.83 (0.55, 1.26) | 0.37 |  | 0.85 (0.53, 1.35) | 0.49 |
|  | Multi- |  | **1.34 (1.09, 1.64)** | **0.01** |  | **1.49 (1.18, 1.89)** | **0.001** |  | 0.98 (0.65, 1.5) | 0.94 |  | 1.05 (0.65, 1.7) | 0.83 |
| SL | Uni- |  | 1.07 (0.87, 1.32) | 0.51 |  | 1.07 (0.85, 1.35) | 0.57 |  | 1.04 (0.71, 1.53) | 0.83 |  | 0.92 (0.59, 1.44) | 0.72 |
|  | Multi- |  | 1.06 (0.86, 1.31) | 0.59 |  | 1.08 (0.85, 1.38) | 0.52 |  | 1.11 (0.75, 1.66) | 0.60 |  | 1.04 (0.66, 1.64) | 0.86 |
| CV | Uni- |  | 1.15 (0.96, 1.39) | 0.14 |  | 1.19 (0.96, 1.47) | 0.12 |  | 1.1 (0.79, 1.55) | 0.57 |  | 1.13 (0.78, 1.65) | 0.51 |
|  | Multi- |  | 1.17 (0.97, 1.42) | 0.11 |  | 1.21 (0.97, 1.51) | 0.10 |  | 1.3 (0.92, 1.84) | 0.14 |  | 1.35 (0.92, 2) | 0.13 |

CI denotes confidence interval, CP Cornell product criterion, CV Cornell voltage criterion, MMSE Mini-Mental State Examination, Multi- multivariate analyses, sHR subdistribution hazard ratio, SL Sokolow-Lyon voltage criterion, Uni- univariate analyses.

For the outcome of cognitive decline, multivariate models were adjusted for age, sex, systolic blood pressure, cholesterol level, body mass index, previous treatment for hypertension, atrial fibrillation, cardiovascular disease, diabetic mellitus, current smoking status, alcohol consumption, and trial treatment (placebo versus antihypertensive treatment), and stratified by education (formal versus non-formal) and diastolic blood pressure (<90mmHg vs ≥90mmHg).

For the outcome of dementia, multivariate models were adjusted for sex, education (formal versus non-formal), systolic blood pressure, cholesterol level, body mass index, atrial fibrillation, cardiovascular disease, diabetic mellitus, current smoking status, alcohol consumption, and trial treatment (placebo versus antihypertensive treatment), and stratified by age (<85 vs ≥85 years old, diastolic blood pressure (<90mmHg vs ≥90mmHg) and previous treatment for hypertension.

# Table e-4 Associations between left ventricular hypertrophy and incident cognitive decline or dementia multinomial logistic regression

|  | | Cognitive decline  Reduction in MMSE score to < 24 or by > 3 points in a year | | | | | | Dementia | | | | | |
| --- | --- | --- | --- | --- | --- | --- | --- | --- | --- | --- | --- | --- | --- |
|  | |  | Analytical sample (n=2645) | |  | Among those who had baseline MMSE of ≥ 24 (1836) | |  | Analytical sample (n=2645) | |  | Among those who had baseline MMSE of ≥ 24 (n = 1836) | |
|  |  |  | Cognitive decline (n=785)  Competing events (n=111) | |  | Cognitive decline (n=569)  Competing events (n=74) | |  | Dementia (n=225)  Competing events (n=164) | |  | Dementia (n=179)  Competing events (n=109) | |
|  |  |  | HR (95% CI) | P value |  | HR (95% CI) | P value |  | HR (95% CI) | P value |  | HR (95% CI) | P value |
| CP | Uni- |  | **1.5 (1.11, 2.03)** | **0.009** |  | **1.5 (1.05, 2.14)** | **0.03** |  | 1.02 (0.61, 1.71) | 0.95 |  | 1.02 (0.57, 1.81) | 0.95 |
|  | Multi- |  | **1.4 (1.02, 1.9)** | **0.04** |  | **1.47 (1.02, 2.12)** | **0.04** |  | 1.02 (0.6, 1.73) | 0.94 |  | 1.08 (0.6, 1.96) | 0.79 |
| SL | Uni- |  | 1.04 (0.77, 1.41) | 0.79 |  | 1.11 (0.78, 1.59) | 0.56 |  | 1.06 (0.66, 1.72) | 0.81 |  | 1.01 (0.58, 1.76) | 0.98 |
|  | Multi- |  | 1.04 (0.76, 1.41) | 0.81 |  | 1.11 (0.77, 1.61) | 0.57 |  | 1.11 (0.68, 1.81) | 0.68 |  | 1.05 (0.59, 1.85) | 0.88 |
| CV | Uni- |  | 1.26 (0.95, 1.66) | 0.11 |  | 1.21 (0.88, 1.68) | 0.25 |  | 1.3 (0.84, 2.01) | 0.23 |  | 1.34 (0.83, 2.15) | 0.23 |
|  | Multi- |  | 1.17 (0.87, 1.56) | 0.30 |  | 1.18 (0.84, 1.66) | 0.34 |  | 1.36 (0.87, 2.13) | 0.18 |  | 1.44 (0.88, 2.38) | 0.15 |

CI denotes confidence interval, CP Cornell product criterion, CV Cornell voltage criterion, MMSE Mini-Mental State Examination, Multi- multivariate analyses, sHR subdistribution hazard ratio, SL Sokolow-Lyon voltage criterion, Uni- univariate analyses.

Multivariate models were adjusted for age, sex, education (formal versus non-formal), systolic and diastolic blood pressure, cholesterol level, body mass index, previous treatment for hypertension, atrial fibrillation, cardiovascular disease, diabetic mellitus, current smoking status, alcohol consumption, and trial treatment (placebo versus antihypertensive treatment).


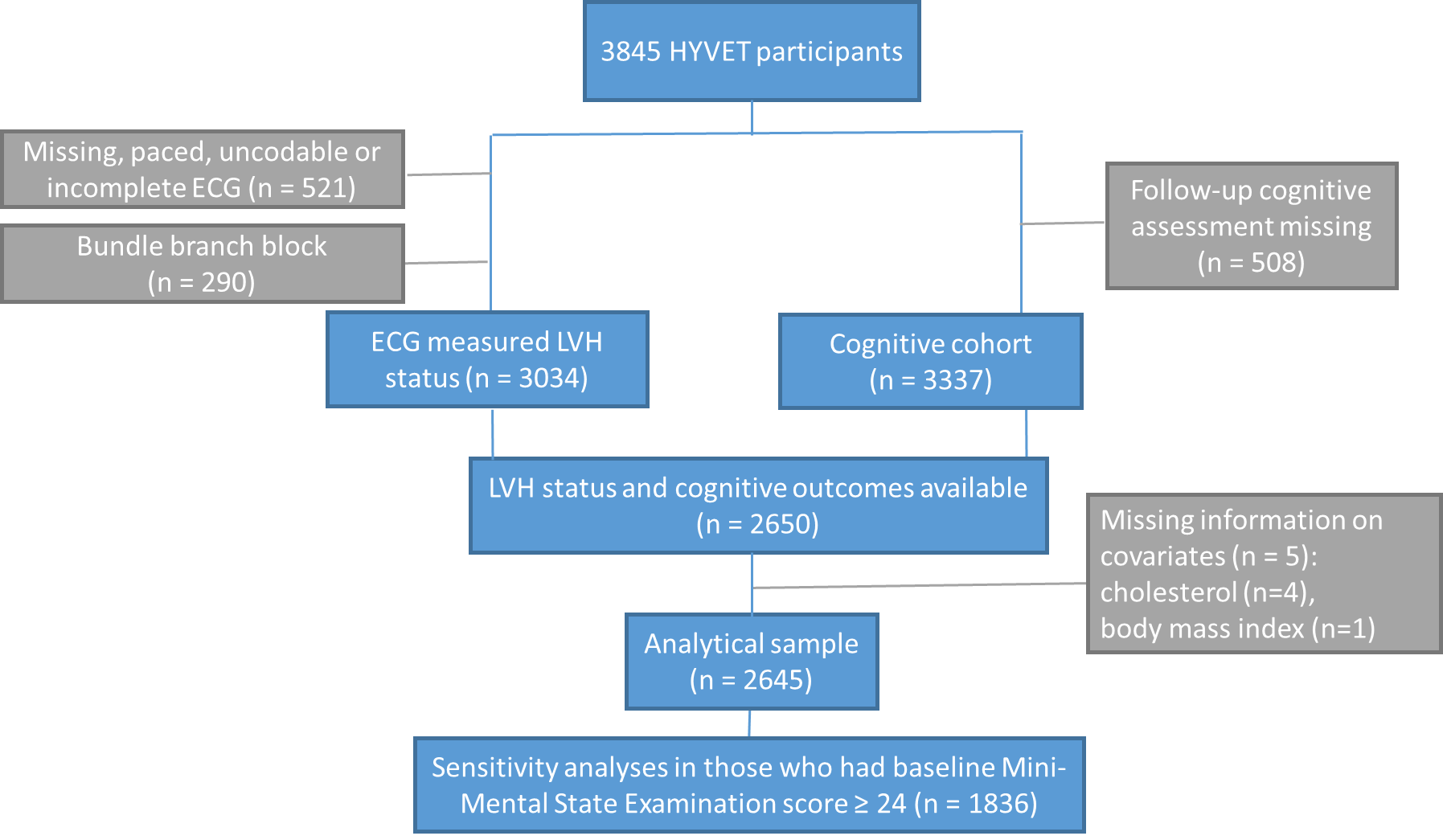


# Figure e-1 Flow chart of the analytical sample and those included in the sensitivity analyses


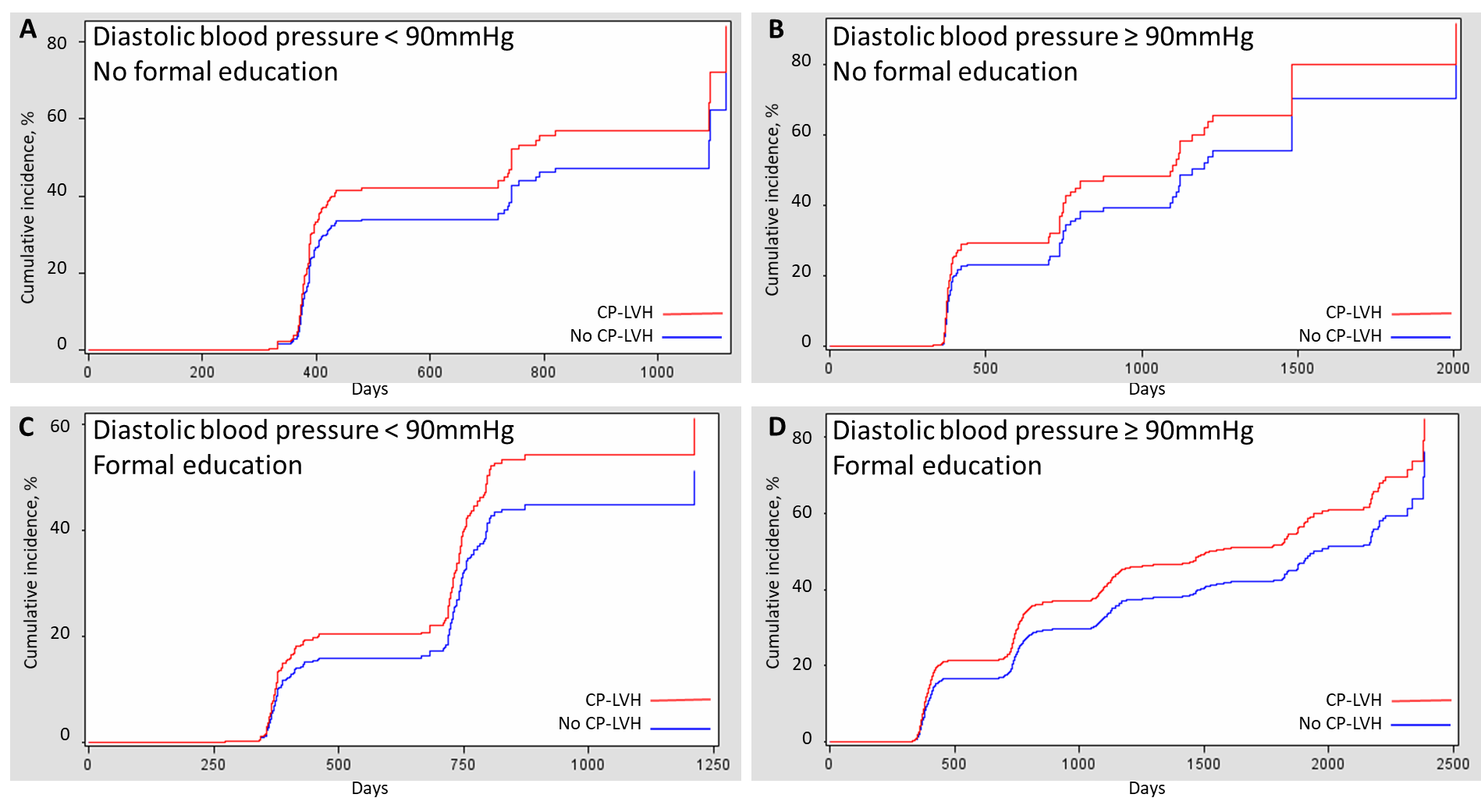


# Figure e-2 Cumulative incidence of cognitive decline by electrocardiography Cornell product criterion defined left ventricular hypertrophy status

CP-LVH denotes Cornell product criterion defined left ventricular hypertrophy. This figure illustrates the differences in cumulative incidence of cognitive decline by left ventricular hypertrophy status in strata (A, B, C and D) for male participants who had mean values of age (83.5 years old), systolic blood pressure (173mmHg), cholesterol level (5.3mmol/L) and body mass index (24.7 kg/m^2^), with no previous treatment for hypertension, no atrial fibrillation, no history of cardiovascular disease, no diabetic mellitus, no alcohol consumption, not currently smoking, and receiving placebo treatment
